# Gender and Side-to-Side Differences of Femoral Condyles Morphology: Osteometric Data from 360 Caucasian Dried Femori

**DOI:** 10.1155/2012/679658

**Published:** 2012-08-30

**Authors:** Ioannis Terzidis, Trifon Totlis, Efthymia Papathanasiou, Aristotelis Sideridis, Konstantinos Vlasis, Konstantinos Natsis

**Affiliations:** Department of Anatomy, Medical School, Aristotle University of Thessaloniki, 54124 Thessaloniki, Greece

## Abstract

The purpose of the present study was to conduct direct measurements in a large sample of dried femori in order to record certain morphometric parameters of the femoral condyles and determine whether there are gender and side differences. Three hundred sixty (Greek) Caucasian dried femori (180 left and 180 right), from 192 males and 168 females, were measured using a digital caliper. The mean age was 67.52 years. The mean bicondylar width of the femur was 8.86 cm ± 0.42 cm in men and 7.85 cm ± 0.30 cm in women (*P* < 0.01). The relative values for the medial condylar depth were 6.11 cm ± 0.34 cm and 5.59 cm ± 0.29 cm (*P* < 0.05); for the lateral condylar depth were 6.11 cm ± 0.33 cm and 5.54 cm ± 0.21 cm (*P* < 0.01); for the intercondylar width were 2.20 cm ± 0.18 cm and 1.87 cm ± 0.10 cm (*P* < 0.001); for the intercondylar depth were 2.78 cm ± 0.16 cm and 2.37 cm ± 0.12 cm (*P* < 0.001). No significant side-to-side difference was observed in any parameter. The femoral condyles differences in anatomy between genders might be useful to the design of total knee prostheses. The contralateral healthy side can be safely used for preoperative templating since there were no significant side differences.

## 1. Introduction

Quantitative anatomy of the distal femur is important for the design of total joint replacement and internal fixation material. Recent studies emphasize on differences between genders and among ethnic groups [1–5]. Preoperative templating for a total knee arthroplasty usually involves the contralateral, healthy side, based on the assumption that there are no side-to-side differences [6]. Furthermore, it has been found that certain osteometric parameters of the femur, such as the femoral intercondylar notch width, differ between genders and are associated with both the volume and the incidence of anterior cruciate ligament (ACL) rupture [7–9]. However, this association has been questioned by other researchers [10, 11]. 

Most morphometric large sample size studies of the distal femur include measurements on radiographs, computerized tomography or magnetic resonance imaging [1, 9, 11, 12]. A study on 1207 dried femora was published recently, where authors performed measurements using a microscribe digitizer for 3D analysis [13]. In the present study, certain osteometric parameters of the femoral condyles were recorded and the existence of gender and side-to-side difference was examined in 360 Caucasian dried femori.

## 2. Materials and Methods

**Figure 1:**
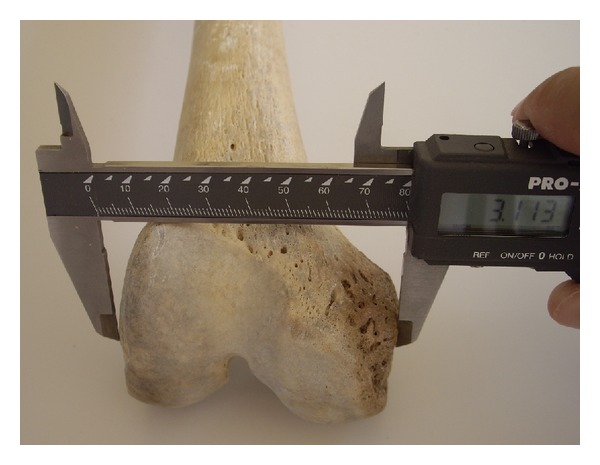
Measurement of the femur bicondylar width.

**Figure 2:**
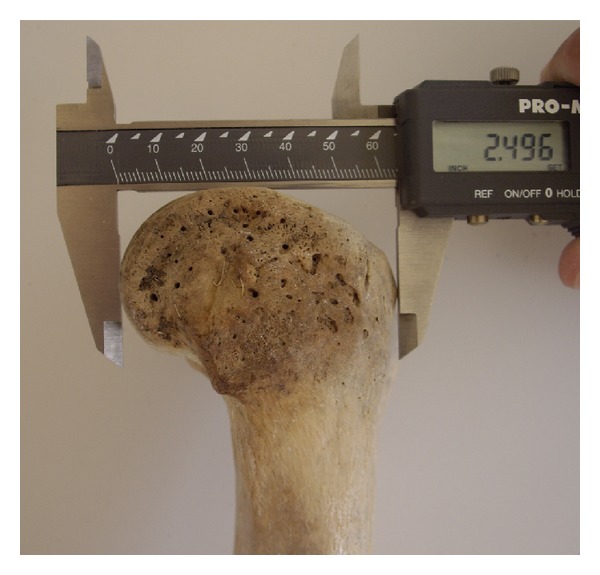
Measurement of the femur medial condylar depth. Similarly the lateral condylar depth was measured.

**Figure 3:**
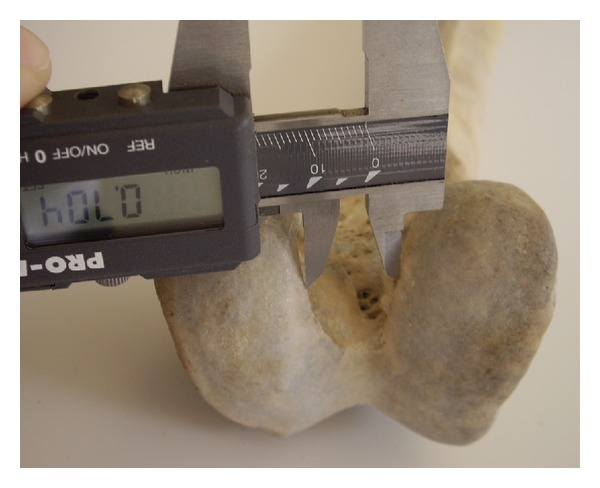
Measurement of the femur intercondylar width.

**Figure 4:**
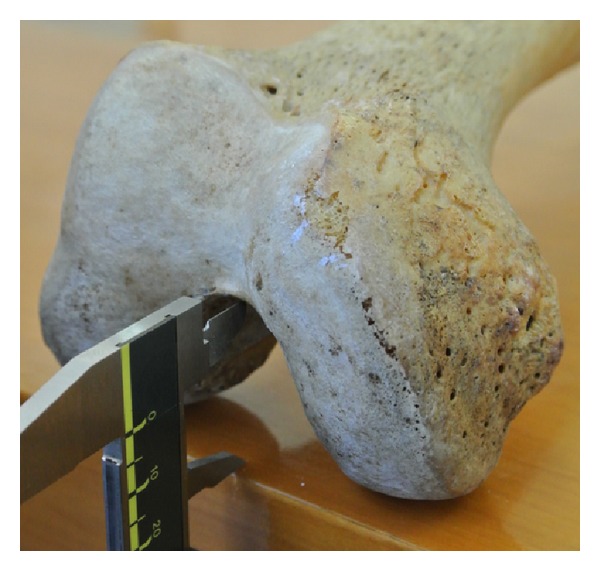
Measurement of the femur intercondylar depth.

The sample consisted of 360 paired dried femori (180 left and 180 right) from 192 males and 168 females. The mean age was 67.52 years (range 40–94 years). Femori that belonged to individuals other than Greeks were excluded. Femori that on gross inspection had evidence of fracture, post-mortem damage or arthritis were excluded from the study, as well. All measurements were performed with a digital sliding caliper. The osteometric parameters were defined as follows: (1) bicondylar width: the maximum distance across the femoral condyles in the transverse plane (Figure 1); (2) medial condylar depth: the maximum anteroposterior diameter of the medial femoral condyle (Figure 2); (3) lateral condylar depth: the maximum anteroposterior diameter of the lateral femoral condyle; (4) intercondylar notch width: the distance between 1/2 the anteroposterior diameter of the lateral surface of the medial femoral condyle and 1/2 the anteroposterior diameter of the medial surface of the lateral femoral condyle (Figure 3); (5) intercondylar notch depth: the vertical distance between the most anterior point of the inferior border of the intercondylar notch and the tangent to the posterior surface of the femoral condyles (Figure 4).

Data analysis was conducted using SPSS for Windows version 18.0. One way ANOVA was used to test for significant differences between genders and sides of the body. A *P*-value less than 0.05 was considered statistically significant. A single author performed all measurements for consistency. Each measurement was repeated three times and the mean value was recorded. Measurement error was assessed for every anatomical parameter according to the method described by White and Folkens for osteometric studies [14]. All measurements were rounded to two decimal places.

## 3. Results

The mean bicondylar width of the femur was 8.39 cm ± 0.63 cm (range, 7.15 cm–9.42 cm). It was 8.86 cm ± 0.42 cm (range, 7.83 cm–9.42 cm) in men and 7.85 cm ± 0.30 cm (range, 7.15 cm–8.20 cm) in women (*P* < 0.01). The mean medial condylar depth was 5.87 cm ± 0.41 cm (range, 5.12cm–6.60 cm). The relative values for the medial condylar depth in men were 6.11 cm ± 0.34 (range, 5.23 cm–6.60 cm) and in women were 5.59 cm ± 0.29 cm (range, 5.12 cm–6.01 cm) (*P* < 0.05). The average lateral condylar depth was 5.85 ± 0.40 (range, 5.11 cm–6.60 cm). It was 6.11 cm ± 0.33 cm (range, 5.32 cm–6.60 cm) in men and 5.54 cm ± 0.21 cm (range, 5.11 cm-5.98 cm) in women (*P* < 0.01). The mean intercondylar width was found 2.05 cm ± 0.22 cm (range, 1.60 cm–2.64 cm). In male femora average value was 2.20 cm ± 0.18 cm (range, 1.89 cm–2.64 cm) and in female femora was 1.87 cm ± 0.10 cm (range, 1.60 cm–2.12 cm) (*P* < 0.001). The intercondylar depth was 2.59 cm ± 0.20 cm on average (range, 2.32 cm–3.10 cm). It was 2.78 cm ± 0.16 cm (range, 2.47 cm–3.10 cm) and 2.37 cm ± 0.12 cm (range, 2.32 cm–2.76 cm) (*P* < 0.001). Data, as well as measurements error values, are summarized in Tables 1, 2, 3, 4, and 5.

## 4. Discussion

In the present study, five morphometric parameters were recorded in dried bones with a direct method using digital sliding caliper. In the literature most anatomic morphometric studies have been conducted with indirect methods including radiography, computerized tomography, magnetic resonance imaging, and 3D modelling. Given the fact that cadaveric material is scarce, these methods offer the advantage of describing anatomy in large samples since they can be performed in living subjects. However, indirect methods have been found to be inaccurate even after correction for magnification, technique, and projection [14–16].

The bicondylar width of the femur was found 8.39 cm±0.63 cm on average. It was significantly (*P* < 0.01) greater in men than in women, but there was no significant difference between the two sides of the body. The bicondylar width is the most frequently measured anatomic parameter of the distal femur. However, there is great variability between studies regarding the definition of measuring points as well as the measurement techniques and the type of sample [1, 4–7, 9–13, 17–19]. As a result, any comparison would provide unreliable conclusions. We measured the bicondylar width of the femur according to the definition of Farrally and Moore which is the maximum distance across the condyles in the transverse plane. [18]. They reported an average of 8.31 cm in 27 Caucasian femori, which is very close to the present study result, and 7.95 cm in 32 Negro femori (*P* < 0.01). Regardless of the measurement method, most studies have demonstrated a greater bicondylar width in men than in women and no statistically significant difference between left and right side [1, 4–7, 9, 10, 12, 13, 17, 19]. 

The mean medial condylar depth of the femur was 5.87 cm ± 0.41 cm. Men had a significantly (*P* < 0.05) greater depth than women. The average lateral condylar depth of the femur in our sample was 5.85 cm ± 0.4 cm and it was also significantly (*P* < 0.01) greater in men than in women. No significant difference was found between the left and right femori for both measurements. In the literature, the condylar depth was uniformly defined as the maximum anteroposterior diameter of each femoral condyle, but differences in measurement techniques and sample material were consistent. Farrally and Moore (1975) reported the “anteroposterior width of femoral condyles”, but they did not clarify which condyle was measured [18]. The greater depth of both femoral condyles in men than in women and the absence of side differences, which were noticed in the present study, are in accordance with most literature studies [1, 4–6, 17, 20, 21]. However, Gillespie et al. [13] measured the medial and lateral flange height and found no difference between men and women.

The bicondylar width as well as the medial and lateral condylar depths of the femur are important parameters for the design of total knee prostheses. Differences in anatomy between genders have led to the design of gender-specific implants. Lateral condyle depth of the femur has been associated with osteoarthritis, but it remains unclear whether the increased depth of the lateral condyle is a predisposing factor or the effect of knee osteoarthritis [20].

The intercondylar width of the femur was 2.05 cm ± 0.22 cm on average, while the mean intercondylar depth was 2.59 cm ± 0.20 cm. Both intercondylar notch dimensions were significantly (*P* < 0.001) greater in men than in women. No significant difference was found between the left and right femori. Intercondylar notch dimensions have a clinical impact since smaller intercondylar notches have been associated with smaller ACL width and more frequent ACL ruptures [7, 9, 20, 22, 23]. However, other studies questioned this association [8, 11, 24, 25]. This controversy led to the publication of morphometric studies of the intercondylar notch with the use of imaging techniques [7–11, 20, 22–24]. Wada et al. reported that there is an association between intercondylar width and knee osteoarhritis but this observation needs further investigation [20].

 The intercondylar width has been studied extensively but this is not the case for the intercondylar depth of the femur. [7–11, 20, 22–25]. Herzog et al. compared intercondylar width measurements obtained with imaging techniques and the direct method [24]. There was no statistical significant difference between measurements obtained with calipers and MRI but there was a significant difference between calipers and X-ray [24]. Based on their observation, we compared our results with those mentioned in the literature and we noticed that they are within the range of the values reported [7–11, 20, 22–25]. The larger intercondylar notch in men and the absence of side-to-side differences, which were found in the present study, have been verified by many authors [7, 9, 10, 22, 23].

In conclusion, in the present study direct measurements of the femoral condyles were conducted in a large sample of Caucasian (Greek) subjects. The differences in anatomy between genders might add to the design of prostheses. However, recent studies have shown that gender differences of distal femur morphometry depend on other morphometric measurements of femur, such as the femur length and width [4]. In the study by Dargel et al. 2011, which included 26 measurements of the knee joint, when gender differences were corrected for differences in femur length, medial-lateral dimensions of knees were still significantly larger in men than in women; however, matched paired analysis did not prove those differences to be consistent [17]. Therefore, they proposed that new implant design might rather take into account interindividual variations in the knee joint anatomy instead of gender-specific variations. Based on the results of the present study, the contralateral healthy side can be used safely for preoperative templating in total knee reconstruction since there was no statistical significant difference. 

## Figures and Tables

**Table 1:** Gender and side distribution of distal femur bicondylar width values (measurement error 1.6%).

	Bicondylar width (CM)				
	Specimens	Mean value	Minimum value	Maximum value	Standard deviation
Gender					
Male	192	8.86	7.83	9.42	0.42
Female	168	7.85	7.15	8.20	0.30

Total	360	8.39	7.15	9.42	0.63

Side					
Left	180	8.37	7.15	9.38	0.63
Right	180	8.41	7.15	9.42	0.62

Total	360	8.39	7.15	9.42	0.63

**Table 2:** Gender and side distribution of femur medial condylar depth values (measurement error 1.1%).

	Medial condylar depth (CM)				
	Specimens	Mean value	Minimum value	Maximum value	Standard deviation
Gender					
Male	192	6.11	5.23	6.60	0.34
Female	168	5.59	5.12	6.01	0.29

Total	360	5.87	5.12	6.60	0.41

Side					
Left	180	5.87	5.12	6.56	0.41
Right	180	5.86	5.12	6.60	0.41

Total	360	5.87	5.12	6.60	0.41

**Table 3:** Gender and side distribution of femur lateral-condylar-depth values (measurement error 1.0%).

	Lateral condylar depth (CM)				
	Specimens	Mean value	Minimum value	Maximum value	Standard deviation
Gender					
Male	192	6.11	5.32	6.60	0.33
Female	168	5.54	5.11	5.98	0.21

Total	360	5.85	5.11	6.60	0.40

Side					
Left	180	5.85	5.11	6.60	0.40
Right	180	5.84	5.12	6.60	0.40

Total	360	5.85	5.10	6.60	0.40

**Table 4:** Gender and side distribution of femur intercondylar width values (measurement error 1.8%).

	Intercondylar width (CM)				
	Specimens	Mean value	Minimum value	Maximum value	Standard deviation
Gender					
Male	192	2.20	1.89	2.64	0.18
Female	168	1.87	1.60	2.12	0.10

Total	360	2.05	1.60	2.64	0.22

Side					
Left	180	2.05	1.62	2.53	0.22
Right	180	2.05	1.60	2.64	0.23

Total	360	2.05	1.60	2.64	0.22

**Table 5:** Gender and side distribution of femur intercondylar depth values (measurement error 1.1%).

	Intercondylar depth (CM)				
	Specimens	Mean value	Minimum value	Maximum value	Standard deviation
Gender					
Men	192	2.78	2.47	3.10	0.16
Female	168	2.37	2.32	2.76	0.12

Total	360	2.59	2.32	3.10	0.20

Side					
Left	180	2.65	2.34	3.10	0.21
Right	180	2.53	2.32	3.02	0.18

Total	360	2.59	2.32	3.10	0.20

## References

[1] Cheng FB, Ji XF, Lai Y (2009). Three dimensional morphometry of the knee to design the total knee arthroplasty for Chinese population. *Knee*.

[2] Chin PL, Tey TT, Ibrahim MYB, Chia SL, Yeo SJ, Lo NN (2011). Intraoperative morphometric study of sex differences in Asian femurs. *Journal of Arthroplasty*.

[3] Urabe K, Mahoney OM, Mabuchi K, Itoman M (2008). Morphologic differences of the distal femur between Caucasian and Japanese women. *Journal of Orthopaedic Surgery*.

[4] Yazar F, Imre N, Battal B, Bilgic S, Tayfun C (2011). Is there any relation between distal parameters of the femur and its height and width?. *Surgical and Radiologic Anatomy*.

[5] Yue B, Varadarajan KM, Ai S, Tang T, Rubash HE, Li G (2011). Gender differences in the knees of Chinese population. *Knee Surgery, Sports Traumatology, Arthroscopy*.

[6] Dargel J, Feiser J, Gotter M, Pennig D, Koebke J (2009). Side differences in the anatomy of human knee joints. *Knee Surgery, Sports Traumatology, Arthroscopy*.

[7] Charlton WPH, John TAS, Ciccotti MG, Harrison N, Schweitzer M (2002). Differences in femoral notch anatomy between men and women. A magnetic resonance imaging study. *American Journal of Sports Medicine*.

[8] Harner CD, Paulos LE, Greenwald AE, Rosenberg TD, Cooley VC (1994). Detailed analysis of patients with bilateral anterior cruciate ligament injuries. *American Journal of Sports Medicine*.

[9] Shelbourne KD, Facibene WA, Hunt JJ (1997). Radiographic and intraoperative intercondylar notch width measurements in men and women with unilateral and bilateral anterior cruciate ligament tears. *Knee Surgery, Sports Traumatology, Arthroscopy*.

[10] Anderson AF, Dome DC, Gautam S, Awh MH, Rennirt GW (2001). Correlation of anthropometric measurements, strength, anterior cruciate ligament size, and intercondylar notch characteristics to sex differences in anterior cruciate ligament tear rates. *American Journal of Sports Medicine*.

[11] Lombardo S, Sethi PM, Starkey C (2005). Intercondylar notch stenosis is not a risk factor for anterior cruciate ligament tears in professional male basketball players: an 11-year prospective study. *American Journal of Sports Medicine*.

[12] Murshed KA, Çiçekcibaşi AE, Karabacakoglu A, Şeker M, Ziylan T (2005). Distal femur morphometry: a gender and bilateral comparative study using magnetic resonance imaging. *Surgical and Radiologic Anatomy*.

[13] Gillespie RJ, Levine A, Fitzgerald SJ (2011). Gender differences in the anatomy of the distal femur. *Journal of Bone and Joint Surgery B*.

[14] White TD, Folkens PA (2000). *Human Osteology*.

[15] Anderson AF, Anderson CN, Gorman TM, Cross MB, Spindler KP (2007). Radiographic measurements of the intercondylar notch: are they accurate?. *Arthroscopy*.

[16] Horsman A, Leung WK, Bentley HB, McLachlan MSF (1977). Effect of rotation on radiographic dimensions of the humerus and femur. *British Journal of Radiology*.

[17] Dargel J, Michael JWP, Feiser J, Ivo R, Koebke J (2011). Human knee joint anatomy revisited: morphometry in the light of sex-specific total knee arthroplasty. *Journal of Arthroplasty*.

[18] Farrally MR, Moore WJ (1975). Anatomical differences in the femur and tibia between negroids and caucasoids and their effects upon locomotion. *American Journal of Physical Anthropology*.

[19] Porter AMW (1995). Analyses of measurements taken from adult femurs of a British population. *International Journal of Osteoarchaeology*.

[20] Wada M, Tatsuo H, Baba H, Asamoto K, Nojyo Y (1999). Femoral intercondylar notch measurements in osteoarthritic knees. *Rheumatology*.

[21] Wanner JA (1977). Variations in the anterior patellar groove of the human femur. *American Journal of Physical Anthropology*.

[22] LaPrade RF, Burnett QM, Daniel DM (1994). Femoral intercondylar notch stenosis and correlation to anterior cruciate ligament injuries. A prospective study. *American Journal of Sports Medicine*.

[23] Souryal TO, Freeman TR, Daniel DM (1993). Intercondylar notch size and anterior cruciate ligament injuries in athletes. A prospective study. *American Journal of Sports Medicine*.

[24] Herzog RJ, Silliman JF, Hutton K, Rodkey WG, Steadman JR (1994). Measurements of the intercondylar notch by plain film radiography and magnetic resonance imaging. *American Journal of Sports Medicine*.

[25] Schickendantz MS, Weiker GG (1993). The predictive value of radiographs in the evaluation of unilateral and bilateral anterior cruciate ligament injuries. *American Journal of Sports Medicine*.

